# Intranasal delivery of plasmids expressing bovine herpesvirus 1 gB/gC/gD proteins by polyethyleneimine magnetic beads activates long-term immune responses in mice

**DOI:** 10.1186/s12985-021-01536-w

**Published:** 2021-03-21

**Authors:** Xing-Bo Liu, Guo-Wei Yu, Xin-Yu Gao, Jin-Long Huang, Li-Ting Qin, Hong-Bo Ni, Chuang Lyu

**Affiliations:** 1 College of Animal Science and Veterinary Medicine, Heilongjiang Bayi Agricultural University, Daqing, 163319 Heilongjiang Province China; 2 Shandong New Hope Liuhe Group Co., Ltd., Qingdao, 266100 China; 3 Qingdao Jiazhi Biotechnology Co., Ltd., Qingdao, 266100 China; 4 College of Veterinary Medicine, Qingdao Agricultural University, Qingdao, 266109 Shandong Province China

**Keywords:** BoHV-1, DNA vaccine, PEI magnetic beads, Nanoparticles, Mucosal immunity

## Abstract

**Background:**

DNA vaccine is one of the research hotspots in veterinary vaccine development. Several advantages, such as cost-effectiveness, ease of design and production, good biocompatibility of plasmid DNA, attractive biosafety, and DNA stability, are found in DNA vaccines.

**Methods:**

In this study, the plasmids expressing bovine herpesvirus 1 (BoHV-1) gB, gC, and gD proteins were mixed at the same mass ratio and adsorbed polyethyleneimine (PEI) magnetic beads with a diameter of 50 nm. Further, the plasmid and PEI magnetic bead polymers were packaged into double carboxyl polyethylene glycol (PEG) 600 to use as a DNA vaccine. The prepared DNA vaccine was employed to vaccinate mice via the intranasal route. The immune responses were evaluated in mice after vaccination.

**Results:**

The expression of viral proteins could be largely detected in the lung and rarely in the spleen of mice subjected to a vaccination. The examination of biochemical indicators, anal temperature, and histology indicated that the DNA vaccine was safe in vivo. However, short-time toxicity was observed. The total antibody detected with ELISA in vaccinated mice showed a higher level than PBS, DNA, PEI + DNA, and PBS groups. The antibody level was significantly elevated at the 15th week and started to decrease since the 17th week. The neutralizing antibody titer was significantly higher in DNA vaccine than naked DNA vaccinated animals. The total IgA level was much greater in the DNA vaccine group compared to other component vaccinated groups. The examination of cellular cytokines and the percentage of CD4/CD8 indicated that the prepared DNA vaccine induced a strong cellular immunity.

**Conclusion:**

The mixed application of plasmids expressing BoHV-1 gB/gC/gD proteins by nano-carrier through intranasal route could effectively activate long-term humoral, cellular, and mucosal immune responses at high levels in mice. These data indicate PEI magnetic beads combining with PEG600 are an efficient vector for plasmid DNA to deliver intranasally as a DNA vaccine candidate.

**Supplementary Information:**

The online version contains supplementary material available at 10.1186/s12985-021-01536-w.

## Background

The development of vaccines based on nucleic acids, i.e., RNA and DNA, is a promising field. DNA vaccine has many advantages, such as cost-effectiveness, ease of design and production, good biocompatibility of plasmid DNA, attractive biosafety, and DNA stability [1]. So far, only a limited number of DNA vaccines have been approved in the veterinary field [2].

Polyethyleneimine (PEI) can be used as a carrier to deliver DNA and RNA in vivo [3]. It has a branch structure containing several amino acids which carry a positive charge. The PEI has been widely used as a transfection reagent in cell assays. The transfection efficiency of PEI is higher than cationic liposome, becoming a preferred embodiment for transfection reagent [4]. The disadvantage of PEI is relatively high toxicity. Thus, the reduction of toxicity and improved transfection efficiency for PEI is a critical direction concerned by researchers. The toxicity of PEI is closely associated with its molecular weight (mw). PEI with mw less than 1 KD has almost no toxicity but has a low transfection efficiency. The toxicity of PEI is elevated as an increase of mw higher than 1 KD. Valeria et al. reported that nanoparticles made of a diversity of toxic chemicals could significantly reduce the toxicity [5]. Nanomaterial has a large specific surface area due to the effects of particle size [6]. A particle size smaller than 500 nm has a better cytophagocytic effect [7, 8]. DNA delivered by superparamagnetic nanomaterials could enhance the transfection efficiency [9]. Thus, the nanoparticles derived from PEI of macromolecule (20 KD) can enhance the transfection efficiency and reduce toxicity.

Bovine herpesvirus type 1 (BoHV-1) is one of the causative pathogens for the bovine respiratory syndrome (BRS) [10]. In spring and autumn, BRS occurrence can lead to infectious bovine rhinotracheitis, abortion of cows, carrying and excreting viruses by diseased cattle, therefore, bringing an enormous loss to the cattle industry. Vaccine immunization is the best approach to prevent this disease. Currently, commercial vaccines include inactivated and gene-deleted vaccines [10]. However, the gene-deleted vaccine's safety is worse than the inactivated vaccine for aged and immunocompromised animals. Additionally, both inactivated and gene-deleted vaccines could not induce a good mucosal immunity, probably due to the muscle injection route.

An in vivo delivery of DNA vaccine into host cells can induce a long-term protein expression at the delivery location and further mediate mucosal, humoral, and cellular immune responses. The gB, gC, and gD proteins of BoHV-1 are immunodominant antigens inducing neutralizing antibodies [11–13]. In this study, the eukaryotic expression plasmids carrying *gB*, *gC*, and *gD* genes of BoHV-1 were used to develop a suitable DNA vaccine for intranasal inoculation. This study aimed to develop a DNA vaccine delivered in mice's respiratory tract for inducing mucosal, humoral, and cellular immunity to provide comprehensive protection against the parent virus challenge.

In the present study, the PEI was coated on the surface of superparamagnetic iron oxide nanoparticles (SPION), namely PEI magnetic beads, to reduce PEI toxicity and increase surface area cytophagocytic effect. This nanoparticle DNA vaccine was prepared by adsorbing antigen-expressing plasmids to PEI magnetic beads. In addition, the polyethylene glycol (PEG) 600 was used as a protective layer to prevent DNA degradation induced by respiratory microbe and mucus and prolong the presence time of DNA in vivo. The prepared nanoparticle DNA vaccine was easy to enter into an organism through the mucosal barrier owing to its small particle size.

## Methods

### Construction of plasmids

The sequences of BoHV-1 *gB*, *gC*, and *gD* genes were obtained from GenBank (Accession No. NC_001847). The genes flanked with restriction sites were synthesized by TSINGKE Biological Technology Company (Beijing, China) and then cloned into eukaryotic expression vector pcDNA-Myc-hisB, resulting in recombinant plasmids pcDNA-*gB*-hisB, pcDNA-*gC*-hisB, and pcDNA-*gD*-hisB. The recombinant plasmids were validated by sequencing.

### Preparation of the DNA vaccine

The eukaryotic expression plasmids pcDNA-*gB*-hisB, pcDNA-*gC*-hisB, and pcDNA-*gD*-hisB were mixed at a mass ratio of 1:1:1. Subsequently, the plasmids were mixed with PEI magnetic beads (Enriching Biotechnology, Shanghai, China) of 50 nm in a maximal adsorption manner. The procedures for identifying the maximal adsorption of PEI magnetic beads and plasmids were: (1) PEI magnetic beads (50 μL) were mixed with 5, 10, 15, 20, 25, and 30 μg plasmid mixture, respectively. The mixture of PEI and plasmids were incubated at room temperature (RT) for 30 min to ensure complete adsorption. (2) The PEI magnetic beads were centrifuged at 13,000 × *g* for 30 min. The OD260 values of DNA in the supernatants were measured. (3) Identification of the maximal DNA adsorption by curve plotting, where the beginning of curve reaching parallel was taken as the maximal adsorption level. (4) The maximal DNA adsorption of magnetic beads was calculated by removing DNA in the supernatant from the total DNA.

The PEI magnetic beads were mixed with 60% of maximal adsorption plasmids to prepare the DNA vaccine. Then, the PEG600 was added into the mixture at a ratio of 1:100 (V/V) to neutralize the positive charge on the surface of PEI and became a protective layer to prevent DNA degradation induced by respiratory mucosa.

### Vaccination protocol

The 8-week-old healthy female Kunming mice were randomly assigned into five groups. The mice were classified into PBS, PEI, DNA, PEI + DNA, and PEI + DNA + PEG groups. In the DNA-containing groups, the mice were vaccinated with 5 μg twice at an interval of 1 month via the intranasal route. In the DNA-free groups, the dose of the drop was kept consistent with DNA vaccine components.

### Immunohistochemistry (IHC)

The lung and spleen were stored in 30% sucrose for 24 h after fixation with 4% PFA. The specimens were sectioned in a cryostat (Microm, Heidelberg, Germany) at 4 μm. IHC was used to detect the expression of viral proteins in the tissues from immunized mice. Briefly, the tissue sections were fixed with absolute ethanol for 30 min. Then, the sections were treated with 1% Triton X-100 for 1 h and followed by treatment of 1% sodium borohydride solution for 30 min at RT. Subsequently, the sections were inoculated with rabbit anti-BoHV-1 polyclonal antibody (prepared in our lab) for 1 h at RT. The sections were rinsed 4 times with PBS-Tween 20 (PBST) and then detected with FITC-conjugated goat anti-rabbit secondary antibody. The expression of viral proteins in each tissue was captured by confocal microscopy (Leica Stellaris 5, US).

### Safety evaluation of DNA vaccine in mice

The prepared DNA vaccine was used to vaccinate mice via the intranasal route. In each group, 8-week-old female Kunming mice were included for anal temperature examination each day after vaccination. Three mice in each group were sacrificed to examine the tissue inflammation, and the sera were collected for detection of phosphorus (PHOS), creatine kinase (CK), aspartate aminotransferase (AST), UREA, and alkaline phosphatase (ALKP) levels from 1 to 5 weeks after vaccination.

### Hematoxylin–eosin (HE) staining

The tissues obtained from the liver, spleen, lung, and kidney were fixed with 4% paraformaldehyde (PFA). The 6 µm thick paraffin tissue sections were prepared for HE staining.

To perform HE staining, the paraffin was removed from tissue sections using dimethyl benzene and then orderly immersed into 100%, 90%, 80%, 70%, and 60% ethanol for 1 min, respectively. Next, the sections were rinsed with pure water and followed by hematoxylin and eosin staining for 3 and 2 min, respectively. Subsequently, the sections were immersed into a 1% hydrochloric acid alcohol solution for differentiation processing. Finally, the stained sections were orderly immersed into 60%, 70%, 80%, 90%, 100% ethanol, and dimethyl benzene for dehydration.

### Flow cytometry

The blood samples (approximately 300 μL) were collected from mouse eyeball using a heparin sodium anticoagulant tube. The triple volume of red blood cell lysis buffer (Solarbio, Beijing, China) was added into the blood for 10 min and then centrifuged at 400 × *g* for 15 min. The supernatant was discarded, and the sedimentary leukomonocytes were resuspended with 300 μL PBS twice. Subsequently, 1 μL FITC-conjugated anti-CD4 antibody (BD, US) and 2 μL PE-conjugated anti-CD8 antibody (BD, US) were incubated with leukomonocytes for 30 min in a dark box. Then, leukomonocytes were rinsed with PBS 3 times. A total of 10,000 cells were counted for calculating the percentages of CD4 and CD8 positive cells using flow cytometry (CytoFLEX S, Beckman Coulter, US).

### Evaluation of humoral immune responses

The enzyme-linked immunosorbent assay (ELISA) was employed to identify anti-BoHV-1 antibody levels. BoHV-1 Cooper strain was purified by ultracentrifugation and then inactivated by ultrasonication (28 kHz, 600 W) for 30 min [14]. The inactivated viruses were used as coating antigens. The ELISA was established in our laboratory. Briefly, 100 ng of antigens were coated in each well of a 96-well plate at 37 °C for 1 h and followed by blocking with 1% BSA at 37 °C for 2 h. The mouse sera were inoculated at 37 °C for 1 h and followed by three washes with PBST. The HRP-conjugated goat anti-mouse IgG secondary antibody (Abcam, UK) was used to detect the anti-BoHV-1 antibody level. The serum antibody level was monitored per week after the second vaccination.

### Evaluation of cellular immune responses

The sera collected from vaccinated mice were used for cytokine detection. According to the manufacturer's instructions, the cytokines IFNγ, IL2, IL4, and IL10 were detected using commercial kits (R&D system, USA).

### Total IgA detection

The supernatant (~ 100 μL) of crushed lung liquid was used for ELISA as described above. The total IgA level was detected with HRP-conjugated goat anti-mouse IgA alpha chain antibodies (Abcam, UK). The difference in IgA level was analyzed among each group.

### Statistics

The statistical data were expressed as mean ± SEM. Statistical analyses were performed using GraphPad Prism 6.01 software (GraphPad Software, Inc.). The difference in biochemical indicators, cytokines, total antibody, total IgA, and CD4/CD8 between DNA vaccine vaccinated and control groups were analyzed by two-way ANOVA post-Sidak's multiple comparison test. The difference in the neutralizing antibody level between DNA and DNA-vaccinated mice was analyzed by unpaired Student's *t*-test. Throughout the manuscript, *P* < 0.05 was taken as the criteria for statistical significance (*: *P* < 0.05, **: *P* < 0.01, ***: *P* < 0.001, ****: *P* < 0.0001, ns: no statistical significance).

## Results

### Characteristics of the DNA vaccine

**Fig. 1:**
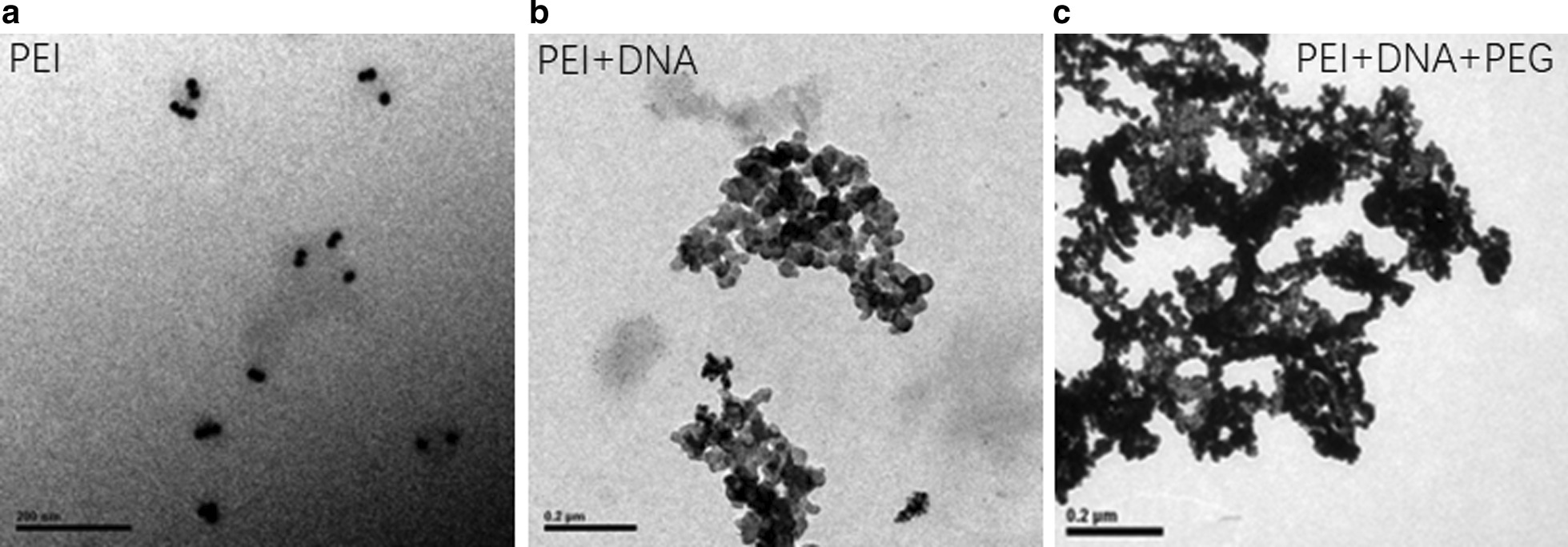
Particle size of PEI magnetic beads adsorbed with DNA and PEG600. **a** The PEI magnetic beads are captured by transmission electron microscopy (TEM). **b** The PEI magnetic beads and DNA form polymers through positive and negative charge attraction. **c** The size of PEI magnetic beads, DNA, and PEG600 agglomerate is further increased. Scale bars indicate 200 nm (**a**–**c**)

The recombinant plasmids pcDNA-*gB*-hisB, pcDNA-*gC*-hisB and pcDNA-*gD*-hisB were mixed at a mass ratio of 1:1:1 and then adsorbed with 30 μL of 50 nm PEI magnetic beads in a 60% of maximal adsorption. The identified maximal DNA adsorption amount of PEI magnetic beads was 25 μg/mL (Additional file 1: Fig. S1). The PEI magnetic beads adsorbed with DNA were coated by double carboxyl PEG600. The particle size of PEI magnetic beads became larger when DNA was adsorbed and formed polymers (Fig. 1a vs. b). The agglomerates' size was further increased after PEG600 involvement, indicating that chain-like PEG was successfully adsorbed with PEI magnetic beads (Fig. 1c). The particle size was measured using a laser particle analyzer. The results showed a duration of 50–70 nm for DNA and PEI magnetic beads polymer and 70–90 nm for DNA, PEI magnetic beads, and PEG600 agglomerate.

### Expression of viral proteins in organs of vaccinated mice

**Fig. 2:**
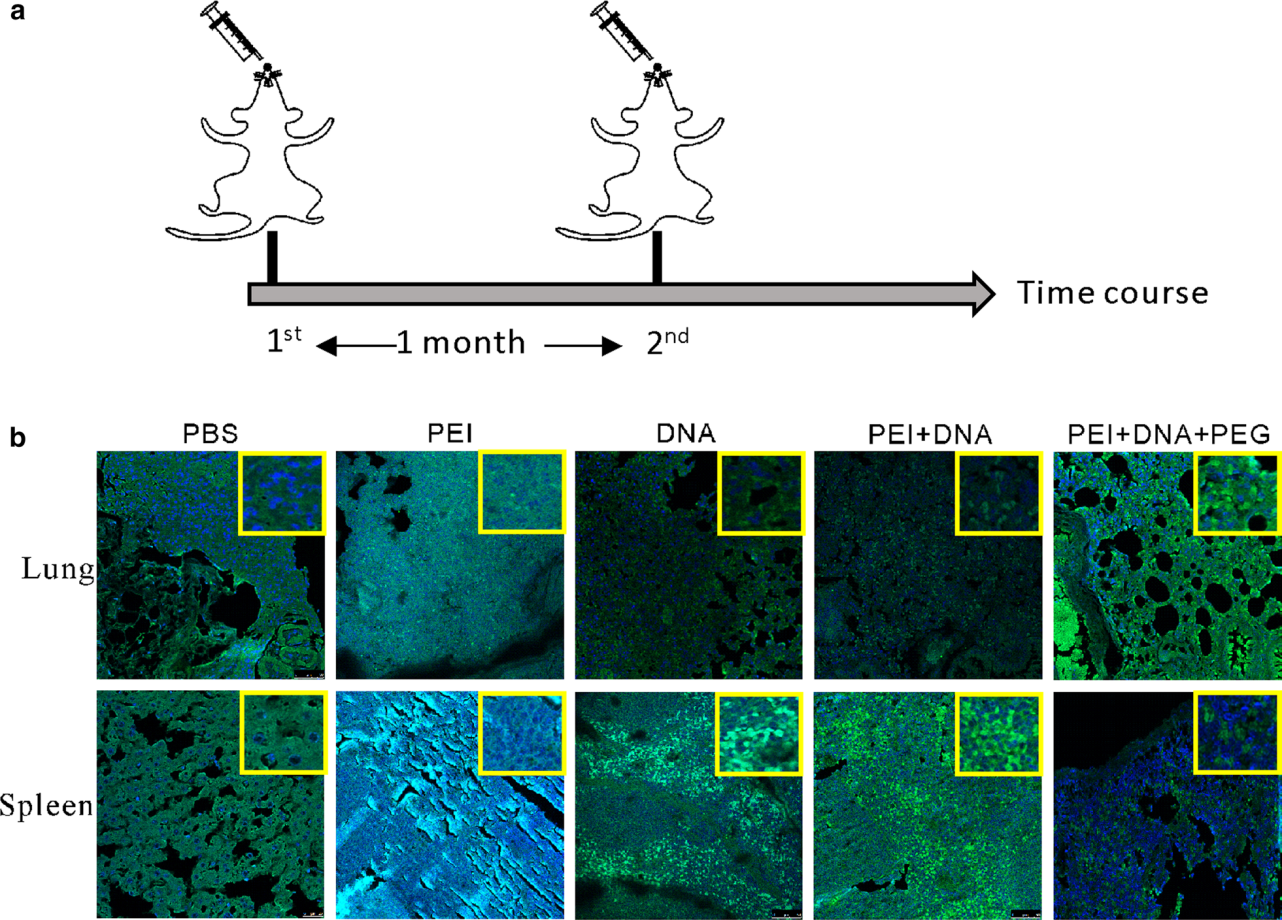
Expression of viral proteins in lung and spleen of mice vaccinated with the DNA vaccine. **a** Schematic shows vaccination protocol. The mice are vaccinated with the DNA vaccine through an intranasal route twice at the 1-month interval. **b** The lung and spleen sections are prepared at the 8th week after the second vaccination. The sections are incubated with anti-BoHV-1 polyclonal antibodies. The expression of viral proteins is identified by IHC. Representative staining was enlarged inside of each micrograph

The mice were intranasally vaccinated with the DNA vaccine twice at the 1-month interval (Fig. 2a). The lung and spleen dissected from vaccinated mice were used for examining the viral protein expression 8 weeks after the second vaccination. The tissue sections were incubated with polyclonal antibodies specifically against BoHV-1. IHC results showed an obvious expression of viral proteins in the lung of PEI + DNA + PEG vaccinated mice, but no viral proteins were detected in PBS, PEI, DNA, or PEI + DNA groups (Fig. 2b). In the spleen, viral proteins' expression was detected in DNA, and PEI + DNA vaccinated mice and much weaker in the PEI + DNA + PEG group, but not in PBS or PEI groups (Fig. 2b).

### Anal temperature examination

**Fig. 3:**
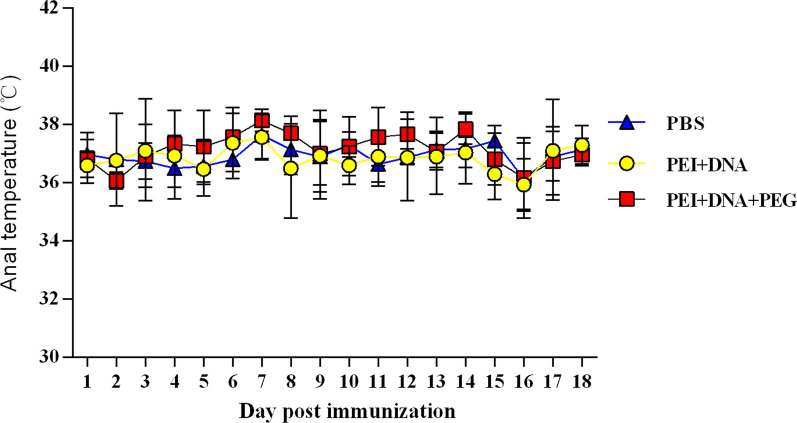
Temperature changes of mice vaccinated with the DNA vaccine. The mice are intranasally vaccinated with the DNA vaccine, and PBS is used as a control. The anal temperature is examined from day 1 to 18 after the second vaccination (n = 3 mice per group on each day)

The anal temperature was examined daily from 1 to 18 days after intranasal vaccination. The results showed a mean temperature duration of 36–38 °C for each group at all examined time points. In addition, no statistical significance was seen among PEI + DNA, PEI + DNA + PEG, or PBS groups on each day (Fig. 3).

### Changes of biochemical indicators in mice vaccinated with DNA vaccine

**Fig. 4:**
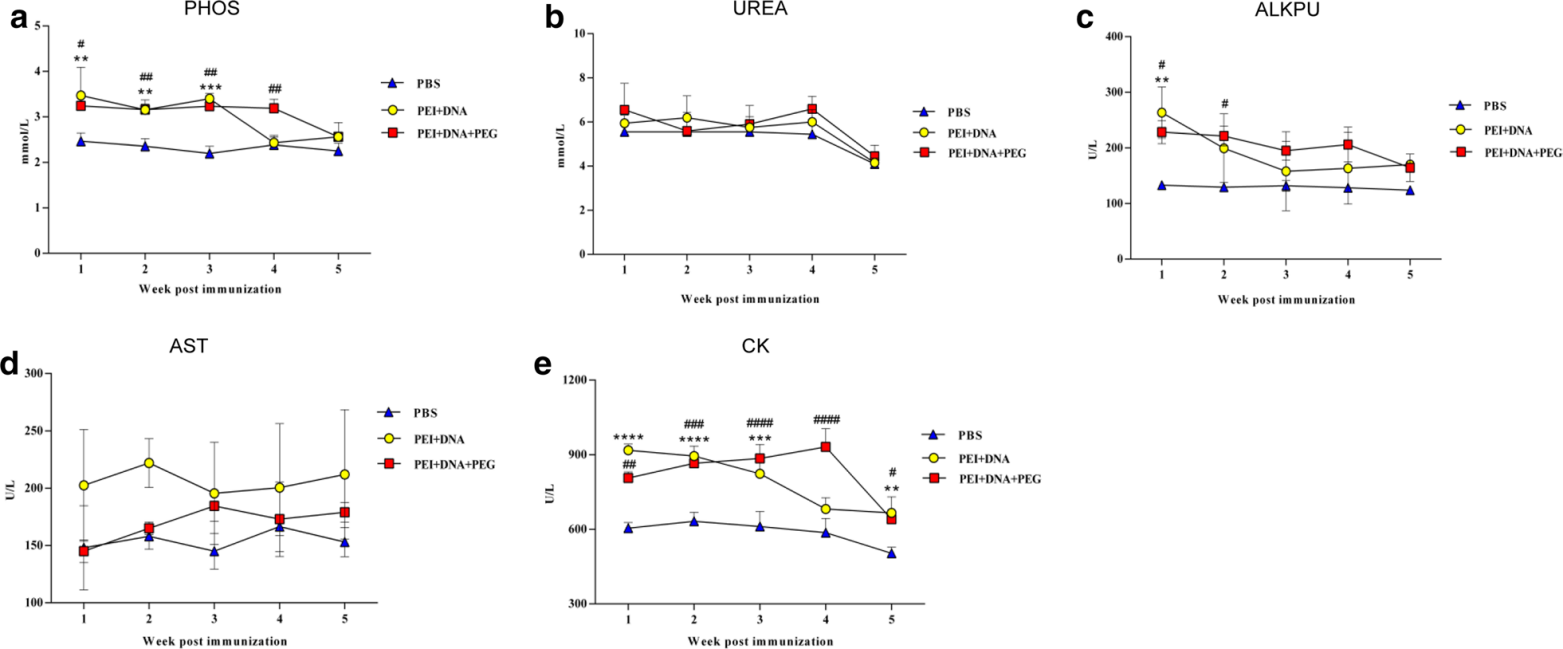
Changes of biochemical indicators in mouse sera after vaccination with DNA vaccine. **a**–**e** The concentration or activity unit of PHOS (**a**), UREA (**b**), ALKPU (**c**), AST (**d**), and CK (**e**) in serum is examined from 1 to 5 weeks after the second vaccination with PEI + DNA and PEI + DNA + PEG (n = 3 mice per group at each time point). The PBS group is used as a control. The symbol “^#^” indicates a comparison between PBS and PEI + DNA + PEG groups, "*" indicates a comparison between PBS and PEI + DNA groups. The statistical analysis is performed by two-way ANOVA post Sidak’s multiple comparison test (^#^*P* < 0.05; ^##,^**P < 0.01; ^###,^***P < 0.001; ^####,^****P < 0.0001)

A series of toxicology-associated biochemical indicators were examined in mice after intranasal inoculation with the DNA vaccine. The PHOS reached a peak level at the first week and started to recover to a normal level at 4–5 weeks in PEI + DNA and PEI + DNA + PEG groups compared to the PBS group (Fig. 4a). In addition, the PHOS level had a faster decrease in the PEI + DNA group than that of the PEI + DNA + PEG group (Fig. 4a). No significant difference was found in the UREA level among PEI + DNA, PEI + DNA + PEG, and PBS groups (Fig. 4b). The ALKPU level was significantly increased in PEI + DNA + PEG and PEI + DNA groups than PBS group at 1 and 2 weeks after vaccination and reduced to a normal level since the third week (Fig. 4c). The AST levels were not significantly different among PEI + DNA, PEI + DNA + PEG, and PBS groups (Fig. 4d). The CK levels were significantly increased in PEI + DNA + PEG and PEI + DNA groups than PBS group, and this increase last at least 5 weeks (Fig. 4e).

### Histopathological changes induced by DNA vaccine

**Fig. 5:**
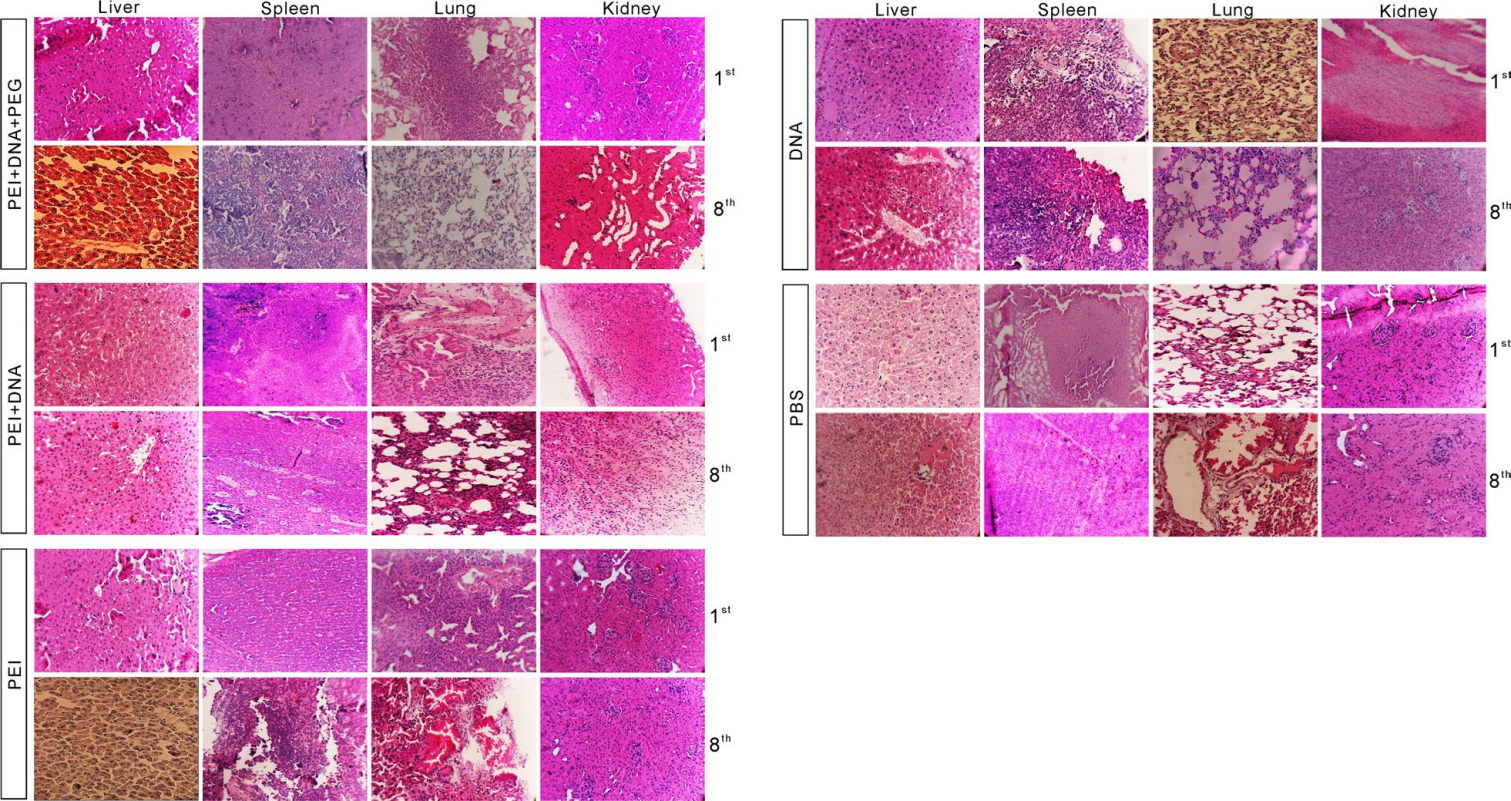
Histopathological examination of mice vaccinated with the DNA vaccine. The mice are vaccinated with the indicated DNA vaccine and its components. The tissue sections derived from the liver, spleen, lung, and kidney are used for histopathological examination at the 1st and 8th week after the second immunization. The PBS group is used as a control

The tissue sections from the mouse liver, spleen, lung, and kidney were subjected to HE staining to evaluate the histopathological changes after DNA vaccine vaccination. The results showed an alveolar thickening in PEI + DNA, PEI + DNA + PEG, and PEI groups compared to DNA and PBS groups at the 1st week after vaccination. However, the lung recovered to a normal state at the 8th week after vaccination (Fig. 5). No histopathological changes were observed in other examined tissues, including the liver, spleen, and kidney (Fig. 5).

### Humoral immune responses induced by DNA vaccine

**Fig. 6:**
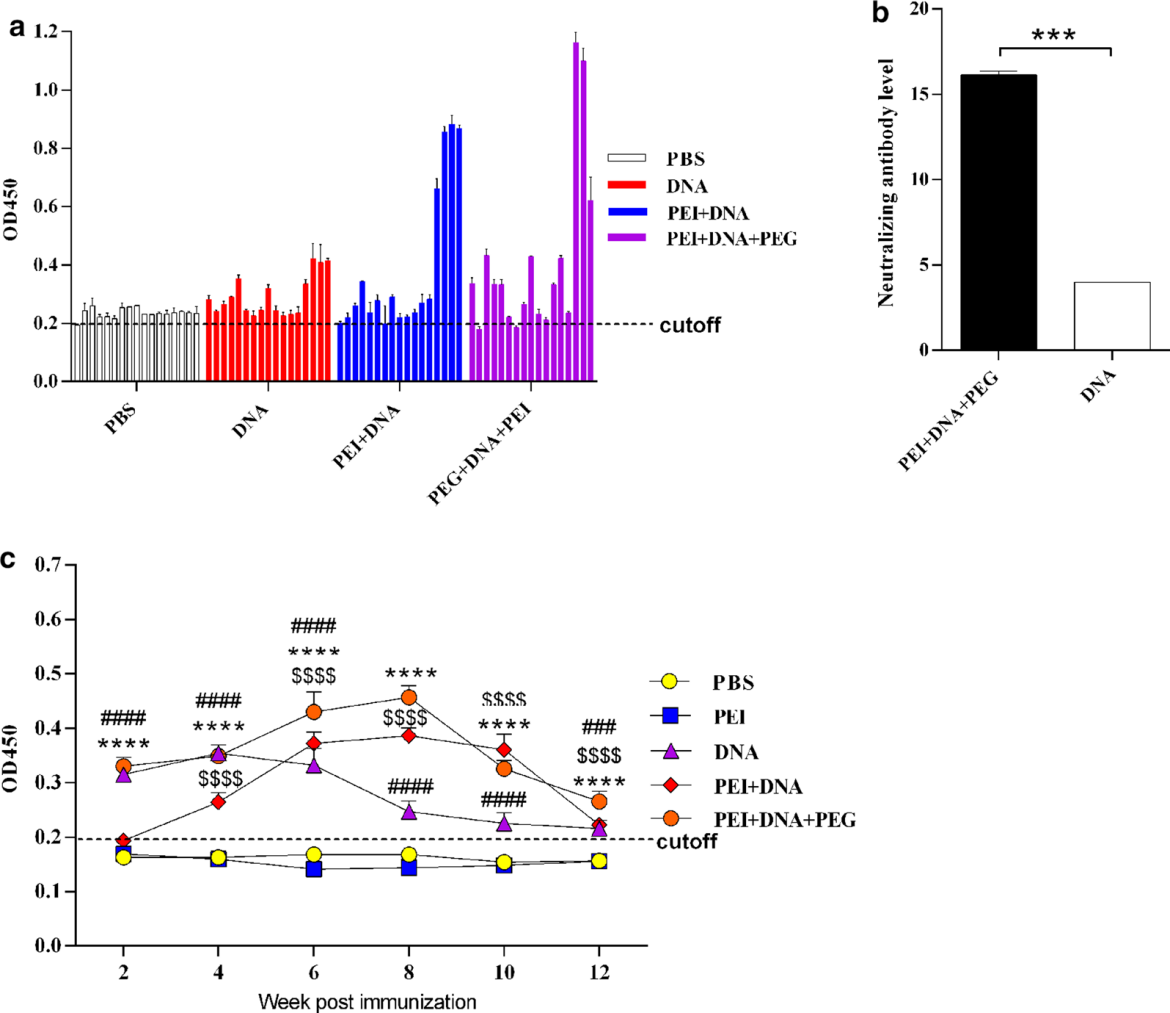
Evaluation of humoral immune responses induced by DNA vaccine in vaccinated mice. **a** Total anti-BoHV1 antibody level in the indicated groups is examined from 1 to 17 weeks after the second vaccination (n = 3 mice per group at each time point). **b** Neutralizing titers of antisera from DNA vaccine vaccinated mice (n = 3 mice per group). The statistical analysis is performed by unpaired Student's *t*-test (***p < 0.001). **c** IgA level is detected in crushed lung liquid (n = 3 mice per group at each time point). The statistical analysis is performed by two-way ANOVA post Sidak’s multiple comparison test (^###^*P* < 0.001; ****,^####,$$$$^*P* < 0.0001)

ELISA was employed to evaluate the total antibody level of anti-BoHV1 in mice vaccinated with the DNA vaccine. A strong increase of antibodies against BoHV-1 was seen in PEI + DNA and PEI + DNA + PEG groups compared to the PBS group at the 15th week after the second vaccination and started to decrease at the 17th week (Fig. 6a). However, the antibody level at the 17th week was still much higher than the earlier time points and control groups. Of note, an increase in total antibody level was also found in the DNA group compared to the PBS group (Fig. 6a).

The titer of neutralizing antibody was significantly higher in the PEI + DNA + PEG group than the DNA group (Fig. 6b). Further, the level of IgA antibodies in lung liquid was detected. Both PEI + DNA and PEI + DNA + PEG groups showed a higher IgA level than PEI and PBS groups from 2 to 12 weeks after vaccination, thus indicating mucosal immunity stimulation (Fig. 6c). Interestingly, the naked DNA vaccination could also promote mucosal immune responses, at short time duration and relatively weak level, to some extent (Fig. 6c).

### Cellular immune responses induced by DNA vaccine

**Fig. 7:**
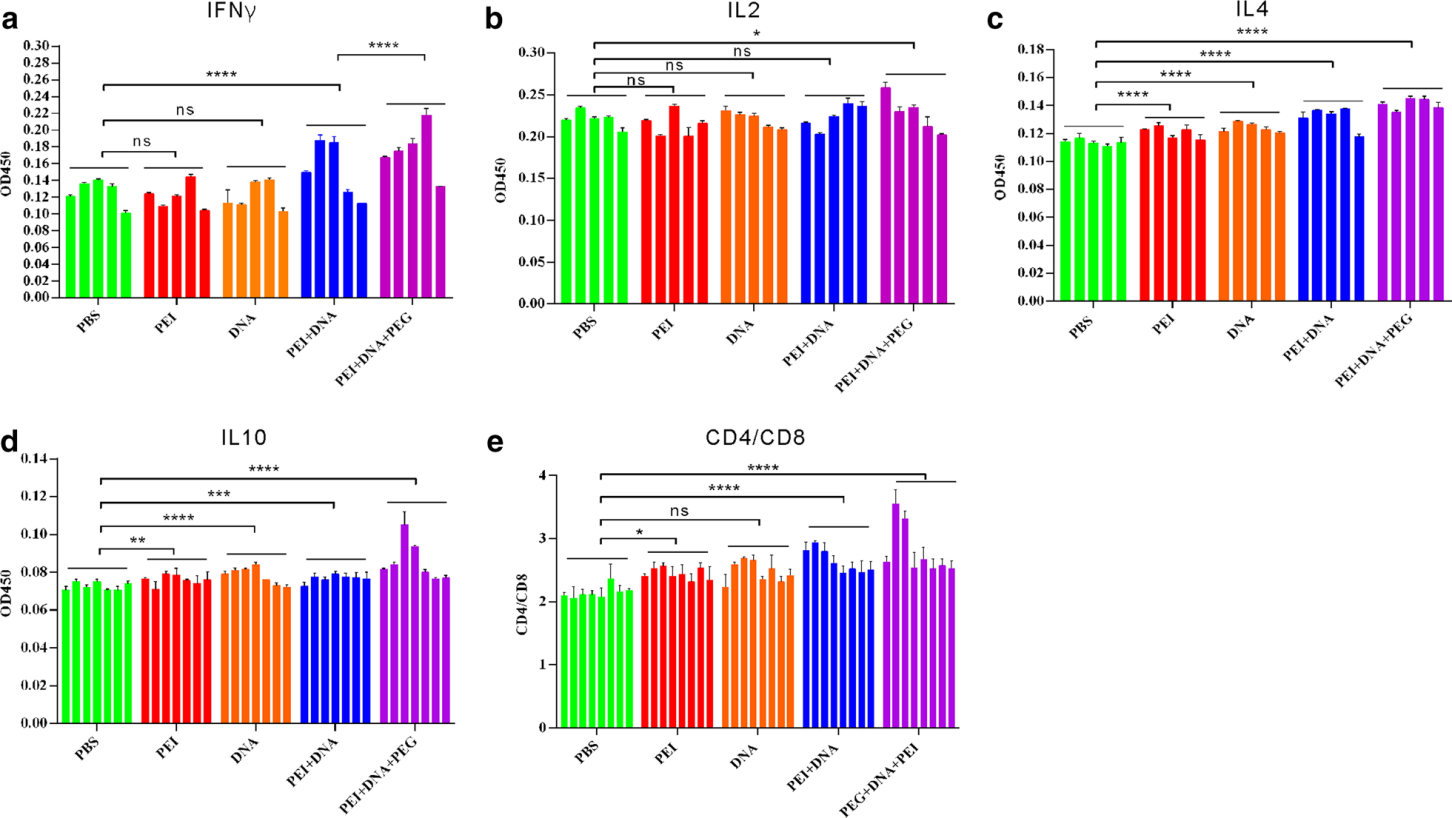
Evaluation of cellular immune responses induced by DNA vaccine in vaccinated mice. **a**–**d** The levels of cytokines IFNγ (**a**), IL2 (**b**), IL4 (**c**), and IL10 (**d**) in sera of mice vaccinated with a DNA vaccine and its components were examined every 2 weeks after vaccination. The PBS group is used as a control. **e** The amount distribution of T lymphocyte subsets is determined in the peripheral blood of mice. The relative level of CD4/CD8 among DNA vaccine and its components in vaccinated mice. The statistical analysis is performed by two-way ANOVA post-Sidak's multiple comparison test (*P < 0.05; **P < 0.01; ***P < 0.001; ****P < 0.0001; ns: no statistical significance)

The expression of cytokines IFNγ, IL2, IL4, and IL10 was detected and compared in the DNA vaccine and its components PEI, DNA, and PEI + DNA vaccinated mice. The PBS group was used as a negative control. Statistical analyses showed that the DNA vaccine in the PEI + DNA + PEG group induced significantly higher expression levels for these cytokines, thus indicating the DNA vaccine could efficiently induce cellular immune responses (Fig. 7a–d). In addition, the ratio of CD4/CD8 was significantly higher in PEI + DNA and PEI + DNA + PEG groups than PBS group, indicating activation of immunocyte (Fig. 7e).

## Discussion

It has been shown that using biodegradable polymeric and magnetic nanoparticles as gene delivery carriers could promote macrophage phagocytosis and activate CD8^+^ T cell reaction [15]. SPION is one of the most popular nano-carriers for gene delivery due to its high surface-to-volume ratio and specific magneticity [16–18]. In addition, the SPION-PEI had a low cytotoxicity [19]. Thus, we prepared the DNA vaccine using PEI magnetic bead as a gene delivery vector in this study.

The PEI magnetic beads have higher efficiency for gene delivery compared to PEI [20]. Using transmission electron microscopy (TEM), greater particle size was seen when the 50 nm PEI magnetic beads were adsorbed with DNA [9]. An involvement of carboxyl PEG600 as an out layer led to a significant increase of particle size and a phenomenon of agglomeration. This observation indicated a fine combination of PEI magnetic beads, DNA and PEG600. The presence of PEI-DNA-PEG agglomerates could prolong the time course for DNA release.

Here, three important genes encoding glycoproteins of BoHV-1 were used as immunogens. The reasons include: (1) these proteins play pivotal roles in viral infection; (2) any glycoprotein is not sufficient to induce full immunoprotection. A previous report indicated the BoHV-1 *gB* gene could activate cytotoxic T cells derived from mice and cattle [21]. Combining immunization of *gD* DNA vaccine and beta-defensin 3 could elevate DNA vaccine efficiency [22]. Meanwhile, Toussaint et al. verified the combination of *gD* and *gC* genes as a DNA vaccine had a better immunoprotection than that of a single gene [13]. Multiple genes combined immunization has a better immune effect than a single gene for BoHV-1 [13].

The immune evaluation of the BoHV-1 vaccine should be performed in the natural host cattle. However, there are several limiting factors affecting this immune evaluation. The limiting factors include: (1) there are no available antibodies for detection of bovine CD4 and CD8 in flow cytometry; (2) there are no commercial kits for detection of bovine cytokines. We had also tried to infect calves with the BoHV-1 strain isolated and kept by our laboratory. However, no clinical symptom was observed after the challenge. The possible explanations for this result could be: (1) the maternal antibodies were present in the calves, probably due to a wide infection of cattle by the BoHV-1 in the field environment; (2) the BoHV-1 strain kept in our laboratory is a low-virulent one. Therefore, it isn't easy to evaluate cattle's immunoreactive processes for the DNA vaccine developed in this study. Despite these limitations, the BoHV-1 vaccine was shown to have uniformity between mice and cattle previously [21]. The mice have also been employed to evaluate the BoHV-1 vaccine, e.g., DNA vaccine, in several studies [23–26]. Thus, we chose mice as model animals to evaluate the BoHV-1 *gB*/*gC*/*gD* genes associated DNA vaccine developed in this study. A further evaluation of cattle is still needed before the vaccine candidate is commercial in the future.

The vaccination of BoHV-1 *gB* gene DNA vaccine by injection could induce mucosal immunity to some extent but could not provide full protection [27]. Thus, the best way to activate mucosal immunity is through the direct mucosal approach. In this study, the nanomaterials were employed to enhance the DNA plasmids' cellular penetration and enhance protein expression at the respiratory tract localization. In turn, long-term expression of these viral glycoproteins could stimulate the mucosal system and enhance immunoprotection duration. The involvement of PEG600 could promote protein expression in the lung, whereas the group without PEG600 exhibited a relatively better safety. Even though no essential difference was observed for DNA vaccine safety in PEG600 presence or not groups, which is probably due to a dramatic decrease of PEI toxicity upon associated with magnetic beads. However, the involvement of PEG600 as a protective layer did prolong the time course of DNA present in the process of respiratory immunity, as shown in the IgA level. An important indicator for DNA vaccine is the immune system's activation and a fine expression of interest protein in target organs. Our data indicated the DNA vaccine could activate both humoral and cellular immunity of a high level compared to the control group. The involvement of PEG600 was an important factor affecting viral protein expression in the lung, which was not seen in the PEG600 absent group.

Collectively, we constructed an efficient DNA vaccine candidate using PEI magnetic beads and PEG600. This DNA vaccine was developed based on nanotechnology and immunology principle. The findings provided a novel strategy for SPION-based DNA vaccine design, which had a potential value for pathogen-specific DNA vaccine development.

## Conclusion

In the current study, we developed a DNA vaccine using a nanoparticle vector. The PEI magnetic bead was used as a DNA vector, and PEG600 was employed to prevent DNA degradation induced by the mucosal system. This DNA vaccine was inoculated via the intranasal route in mice. Experimental results indicated this DNA vaccine was safe in vivo and could activate good humoral, cellular, and mucosal immune responses.

## Supplementary Information

**Additional file 1.** **Fig. S1**: Identification of the maximal adsorption of PEI magnetic beads with plasmids. The maximal adsorption of PEI magnetic beads is 25 μg/mL.

## Data Availability

The corresponding authors would supply the data and materials upon required.

## Abbreviations

BoHV-1: Bovine herpesvirus 1
PEG: Polyethylene glycol
PEI: Polyethyleneimine
BRS: Bovine respiratory syndrome
SPION: Superparamagnetic iron oxide nanoparticles
PBS: Phosphate-buffered saline
IHC: Immunohistochemistry
PBST: PBS-Tween 20
PHOS: Phosphorus
CK: Creatine kinase
AST: Aspartate aminotransferase
ALKP: Alkaline phosphatase
HE: Hematoxylin–eosin
ELISA: Enzyme-linked immunosorbent assay
